# In Search for Reasons behind *Helicobacter pylori* Eradication Failure–Assessment of the Antibiotics Resistance Rate and Co-Existence of *Helicobacter pylori* with *Candida* Species

**DOI:** 10.3390/jof9030328

**Published:** 2023-03-07

**Authors:** Ana Bačić, Vladimir Milivojević, Isidora Petković, Dušan Kekić, Ina Gajić, Branislava Medić Brkić, Dušan Popadić, Tomica Milosavljević, Mirjana Rajilić-Stojanović

**Affiliations:** 1 Department of Biochemical Engineering and Biotechnology, Faculty of Technology and Metallurgy, University of Belgrade, Karnegijeva 4, 11000 Belgrade, Serbia; 2 Clinic for Gastroenterology and Hepatology, University Clinical Centre of Serbia, 11000 Belgrade, Serbia; 3 Faculty of Medicine, University of Belgrade, 11000 Belgrade, Serbia; 4 Institute for Microbiology and Immunology, Medical Faculty, University of Belgrade, 11000 Belgrade, Serbia; 5 Institute for Pharmacology, Clinical Pharmacology and Toxicology, Medical Faculty University of Belgrade, 11000 Belgrade, Serbia; 6 Euromedic General Hospital, 11000 Belgrade, Serbia

**Keywords:** *Helicobacter pylori*, *Candida* spp., gastric microbiota, antibiotic resistance

## Abstract

*Helicobacter pylori* eradication is characterized by decreasing successful eradication rates. Although treatment failure is primarily associated with resistance to antibiotics, other unknown factors may influence the eradication outcome. This study aimed to assess the presence of the antibiotics resistance genes in *H. pylori* and the presence of *Candida* spp., which are proposed to be endosymbiotic hosts of *H. pylori*, in gastric biopsies of *H. pylori-*positive patients while simultaneously assessing their relationship. The detection and identification of *Candida* yeasts and the detection of mutations specific for clarithromycin and fluoroquinolones were performed by using the real-time PCR (RT-PCR) method on DNA extracted from 110 gastric biopsy samples of *H. pylori*-positive participants. Resistance rate to clarithromycin and fluoroquinolone was 52% and 47%, respectively. Antibiotic resistance was associated with more eradication attempts (*p* < 0.05). *Candida* species were detected in nine (8.18%) patients. *Candida* presence was associated with older age (*p* < 0.05). A high rate of antibiotic resistance was observed, while *Candida* presence was scarce, suggesting that endosymbiosis between *H. pylori* and *Candida* may not be a major contributing factor to the eradication failure. However, the older age favored *Candida* gastric mucosa colonization, which could contribute to gastric pathologies and microbiome dysbiosis.

## 1. Introduction

*Helicobacter pylori* is a Gram-negative, microaerophilic flagellated bacterium which is highly adjusted to the generally unfavorable conditions for microbial growth present in the human stomach. *H. pylori* is embedded in the gastric mucosa, where it locally adjusts the pH to a neutral value due to urease activity [1,2]. It is estimated that nearly 50% of the human population carries *H. pylori* in the stomach, with colonization prevalence ranging from 35% in developed countries to a strikingly high prevalence of 80% in some developing countries [3,4]. Although around 70% of the *H. pylori* carriers remain asymptomatic, *H. pylori* colonization is associated with several gastric pathologies, including gastritis, peptic ulcers, non-cardiac gastric adenocarcinoma and gastric mucosa-associated lymphoid tissue (MALT) lymphoma [1,5]. Today, *H. pylori* is recognized as a type 1 carcinogenic agent, responsible for ~90% of gastric cancer cases [1,6].

Confirmed infection with *H. pylori* is treated with antibiotics, and according to the WHO Global guidelines, triple therapy, comprising of clarithromycin, amoxicillin and a proton-pump inhibitor (PPI) is the most commonly used first-line treatment for the eradication of *H. pylori* in low-resistance regions, but according to new *Maastrich VI* guideline treatment with PPI-clarithromycin-containing triple therapy without prior susceptibility testing should be abandoned when the clarithromycin resistance rate in the region is more than 15% [7,8]. Data from the European Registry reveals that only quadruple therapies lasting at least 10 days are able to achieve over 90% eradication rates [9]. The data for second line treatment revealed that empiric second-line regimens including 14-day quinolone triple therapies, 14-day levofloxacin–bismuth quadruple therapy, 14-day tetracycline–bismuth classic quadruple therapy and 10-day bismuth quadruple therapy (as a single capsule) provided optimal effectiveness [10]. However, the worldwide increase in the prevalence of antibiotic-resistant *H. pylori* strains has compromised the effectiveness of antibiotic therapy and decreased the rate of successful eradication, with the alarming increase of multidrug-resistant *H. pylori* strains [8,11,12,13]. Recently published data on antimicrobial susceptibility of *H. pylori* from multiple centers across the USA and Europe, revealed an alarmingly high rate of clarithromycin resistance with a rate >15% in every region of the USA [14].

In addition to antibiotic resistance, *H. pylori* eradication success is also influenced by a number of other factors. It is well established that PPIs enhance the effect of antibiotics by increasing the gastric pH and ultimately *H. pylori* eradication. Their effect is under influence of the drug-metabolizing enzyme CYP2C19 and its gene polymorphism [15]. Moreover, several other mechanisms for the *H. pylori* resistance to the applied antibiotic therapy were proposed. Studies have shown that bacteria upon establishing certain cell density have the ability to produce extracellular substances, allowing them to form a biofilm. Biofilm provides a self-protective matrix that can make the bacteria up to 1000 times more resistant to antibiotics, and this mechanism could also potentially contribute to the failure of *H. pylori* eradication efforts [16]. Furthermore, it has recently been shown that *H. pylori* behaves as a facultative intracellular bacterium, found inside eukaryotic cells including human gastric epithelial cells, and macrophages and dendritic immune cells [17,18]. Interestingly, the presence of *H. pylori* inside the *Candida* yeasts’ vacuoles has been confirmed recently by fluorescence microscopy and *H. pylori*-specific genes and proteins have been isolated from the yeast cells [17,18,19,20]. Internalization of the *H. pylori* inside the *Candida* yeast cells has been characterized as a protective adaptation to stressful conditions, including changes in pH values, antibiotics treatments, and nutrient-deficient environment [16,21,22,23]. This endosymbiotic relationship has been demonstrated in samples from various body sites, including oral, gastric, and vaginal samples and different food sources [17]. In contrast to *H. pylori*, *Candida* cells are resistant to environmental stress and can survive temperatures above 40 °C and a pH in the range between 2 and 10 [16,17,18,19]. Several studies have shown that *Candida* colonization of the gastric mucosa is associated with several gastric pathologies. Moreover, the co-existence of *Candida* species and *H. pylori* has been confirmed in several studies and a synergistic relationship between these two microorganisms in the pathogenesis of gastric diseases has been suggested [16,24]. Based on this long-standing relationship between the presence of *Candida* and *H. pylori*, we hypothesized that *Candida* presence in the gastric compartment could contribute not only to *H. pylori* pathologies but also to the resistance to the applied antibiotic therapy.

For a long time, *H. pylori* has been considered the only true inhabitant of the human stomach. However, despite the harsh environmental conditions, the existence of a thriving and dynamic gastric microbial community has been proven [24,25,26,27]. It is estimated that the stomach is inhabited by 10^2^–10^4^ colony-forming units (*CFU*)/mL [28]. In addition to the bacterial communities, members of the fungal microbiota have also been identified in gastric biopsy samples, in the range of 0–10^2^ *CFU*/mL [24,25,28]. *Candida* represents the most frequently isolated genus of yeasts, with *C. albicans as* the most frequently identified species [16,21,29]. As previously mentioned, we hypothetitzed that endosymbiosis between *Candida* and *H. pylori* could facilitate the *H. pylori* resistance to eradication treatment, as *Candida* yeasts could serve as reservoirs for an easier transmission [16]. In this setting, a high prevalence of *Candida* infection might contribute to an increase in *H. pylori* infection rates and might contribute to the challenges of its eradication.

To test the hypothesis in the present study we used molecular methods to test the frequency of concurrent infections with *H. pylori* and *Candida* fungi on a cohort of 110 *H. pylori-*positive patients. In addition to *Candida* detection and species level identification, any possible relationship between *Candida* presence in different histopathological categories of biopsies, demographic characteristics of patients, number of eradications and the type of antibiotic resistance mutation detected in *H. pylori* strains, was assessed.

## 2. Material and Methods

### 2.1. Patients

A total of 123 patients with symptoms of dyspepsia recruited at the University Clinical Centre of Serbia were enrolled in the study. During gastroscopy, gastric tissue biopsy samples from the patients’ stomach antrum, angular incisura and corpus were obtained according to the updated Sydney classification of gastritis [30]. The gastric samples were collected between September 2019 and 2021. The *H. pylori*-positive test was the inclusion criteria for the study. Demographic and clinical data of *H. pylori*-positive patients were recorded. Histological examinations were carried out in the Department of Pathohistology of the University Clinical Centre of Serbia. A semi-quantitative categorization of histopathological findings was performed and atrophy, inflammation and cellularity scores were assessed.

### 2.2. Ethical Statement

The study was approved by the Ethics Committee of the University Clinical Centre of Serbia (number 788/11). All patients were informed regarding the aim of the study and provided written informed consent in accordance with the World Medical Association Declaration of Helsinki.

### 2.3. DNA Extraction

Isolation of DNA from biopsies of the gastric mucosa was performed using the commercial kit, QIAmp DNA Mini (Qiagen, Hilden, Germany), according to the manufacturer’s instructions.

### 2.4. H. pylori Detection and Resistance

*H. pylori* was detected using the Giemsa stain (Giemsa’s stain solution of Muto Pure Chemicals Co., Ltd., Tokyo, Japan) on the mucosal surface during the histology examination and by molecular testing. The presence of mutations in genes related to antibiotic resistance was detected for the following genes involved in the *H. pylori* resistance mechanisms: *23S, gyrA*, *gyr87* and *gyr91.*

Molecular detection of *H. pylori* and resistance to clarithromycin and fluoroquinolones was performed using the GenoType HelicoDR kit (Hain Lifescience, Nehren, Germany), according to the manufacturer’s instructions. Briefly, the highly specific region, as well as regions of DNA whose mutations lead to resistance to clarithromycin (CLA) and fluoroquinolones (gyrA), were amplified by RT-PCR, using biotin-labelled primers. Amplified products were then denatured and hybridized using specific oligonucleotides (probes) that are complementary to sequences characteristic of wild alleles (WT probes) and sequences of mutated alleles (MUT probes). Reverse hybridization was performed using a TwinCubator at a temperature of 45 °C. A denaturation solution was added to the amplified product (20 μL), after which the remaining steps were performed according to the manufacturer’s protocol (Hain Lifescience, Nehren, Germany) [31].

### 2.5. Candida *spp.* Detection

*Candida* yeasts were detected using the RT-PCR protocol described in a study by Zhang et al. [32]. In short, extracted DNA from gastric biopsy samples was amplified using Mastercycler^®^ ep Gradient S realplex RT-PCR system (Eppendorf) and QuantiNovaTM SYBR^®^ Green PCR Kit (Qiagen). For *Candida* yeasts detection, the following primers were used: 5.8S-1F5′-*CAA CGG ATC TCT TGG TTC TC-3*′ and 28S-1R 5′-*CGG GTA GTC CTA CCT GAT TT-3*′ [32]. *Candida* species were identified based on the obtained and the reference melting curve profiles. Samples were tested for the presence of several *Candida* species, including *C. albicans, C. tropicalis* and *C. parapsilosis*. For positive controls, *C. albicans* ATCC24433, *C. tropicalis* ATCC23705 and *C. parapsilosis* ATCC24574 were used. Melting curve analysis was performed by gradually increasing the temperature from 65°C to 95 °C at the rate of 1.5 °C/min. The species-specific melting temperatures used for the identification of *Candida* species are shown in Table 1.

*Candida* yeasts were detected using the RT-PCR protocol described in a study by Zhang et al. [32]. In short, extracted DNA from gastric biopsy samples was amplified using Mastercycler^®^ ep Gradient S realplex RT-PCR system (Eppendorf) and QuantiNovaTM SYBR^®^ Green PCR Kit (Qiagen). For *Candida* yeasts detection, the following primers were used: 5.8S-1F5′-*CAA CGG ATC TCT TGG TTC TC-3*′ and 28S-1R 5′-*CGG GTA GTC CTA CCT GAT TT-3*′ [32]. *Candida* species were identified based on the obtained and the reference melting curve profiles. Samples were tested for the presence of several *Candida* species, including *C. albicans, C. tropicalis* and *C. parapsilosis*. For positive controls, *C. albicans* ATCC24433, *C. tropicalis* ATCC23705 and *C. parapsilosis* ATCC24574 were used. Melting curve analysis was performed by gradually increasing the temperature from 65° to 95 °C at the rate of 1.5 °C/min. The species-specific melting temperatures used for the identification of *Candida* species are shown in Table 1.

### 2.6. Statistical Analysis

Statistical analyses were performed in order to assess the relationship between *Candida* colonization, presence of *H. pylori* gene mutations related to antibiotic resistance and demographic data of patients, as well as the number of eradications and the histopathological scores of biopsies. Non-parametric methods, Chi-square and Fisher’s Exact tests were used for data analysis with a level of significance of *p* < 0.05. The data were collected and analyzed using SPSS version 26.

## 3. Results

### 3.1. Patients Enrolled and Baseline Characteristic

Out of 123 patients in the cohort, biopsy samples of 13 patients were not analyzed in detail due to the negative *H. pylori* detection results or incomplete information regarding histopathological criteria. For 110 confirmed *H. pylori*-positive patients, the demographic and clinical data were recorded. The age of participants in the analyzed cohort ranged between 21 and 91 years, with a median age of 56 years. The most reported gastric pathologies included gastritis (*n* = 74; 67.3%), dyspepsia (*n* = 19; 17.3%), peptic or duodenal ulcers (*n* = 11; 10.0%) and gastroesophageal reflux disease (GERD) (*n* = 11; 10.0%). Gastric biopsies of the participants were investigated, and samples were categorized according to cellularity, inflammatory and atrophy scores.

### 3.2. Real-Time PCR Detection of Candida Species in DNA Isolated from Gastric Biopsies

*Candida* species were detected and identified using described real-time PCR method in nine (8.18%) participants (Table 1). At the species level, *C. albicans* was most frequently detected as it was present in five patients (representing 55.6% of *Candida-*positive samples), while *C. tropicalis* and *C. parapsilosis* were present in two (22.2%) samples each.

### 3.3. Statistical Assessment of Association between Candida Presence in Gastric Biopsies and Demographic, Clinical and Histological Parameters

Participants were classified into *Candida*-positive and *Candida*-negative groups. Statistical analyses were performed by assessing the relationship between the distribution of *Candida*-positive and *Candida*-negative patients following different histopathological categories (cellularity levels, inflammatory activity, and observed rate of atrophy), demographic categories (age, gender), number of eradication and the type of antibiotic resistance mutation detected in *H. pylori* strain (Table 2).

A significantly older age was detected in the *Candida*-positive group, with a median age of 66 years, compared to a median of 55 years among the *Candida*-negative patients (*p* < 0.05). Moreover, all *Candida*-positive patients were older than 50 years, compared to the *Candida*-negative group in which 59 participants (58.4%) were >50 years old.

**Figure 1:**
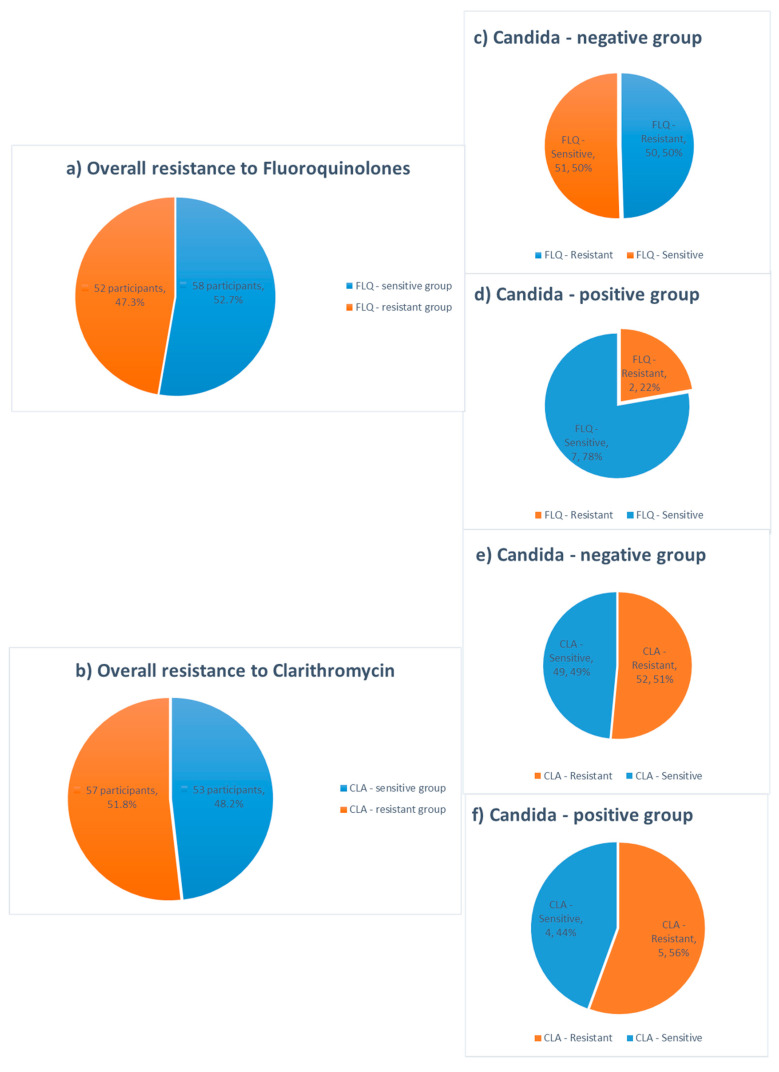
Overall resistance rates to fluoroquinolones and clarithromycin detected by PCR and antibiotic resistance rates in *Candida*-negative and *Candida*- positive groups (**a**–**f**).

The results of antibiotic resistance testing indicate high resistance rates to both clarithromycin and fluoroquinolone drugs in *Candida*-positive and *Candida*-negative groups, with no statistically significant difference between them, as presented in the Table 2 and Figure 1. The most commonly noted mutations included 23S and gyrA mutations, with more than 50% of patients possessing 23S gene mutations in both the *Candida*-positive (55.6%) and the *Candida*-negative groups (50.5%).

The statistical analysis revealed absence of a significant association between the presence of *Candida* species and any of the assessed histopathological categories, number of eradications, gender and the presence of *H. pylori* mutations (*p* > 0.05). The cellularity score of 2 was most frequently noted in both groups. Biopsy inflammatory scores of 1 and 2 were the most frequently observed, with the highest rate of inflammatory activity score of 1 in both *Candida*-positive and *Candida-*negative groups. Regarding the atrophy assessment, atrophy scores 0 and 1 were most commonly observed, with the majority of patients having an atrophy score of 1, 51% and 66.7% for *Candida-*negative and *Candida*-positive patients, respectively (Table 2).

Concerning the pathologies, among *Candida*-positive participants, the most commonly diagnosed gastrointestinal pathologies included GERD, which was detected in three (33.3%) patients, followed by dyspepsia in two (22.2%), gastroduodenitis in two (22.2%) and gastritis and duodenal ulcer in one patient each (11.1%). Other documented pathologies including sideropenic anemia, esophageal varices and diaphragmatic hernia were absent among *Candida*-positive patients. The most common diseases diagnosed in the *Candida*-negative group included gastritis, which was diagnosed in 39 patients, followed by gastroduodenitis diagnosed in 20 patients. Diagnosis of gastritis or duodenitis was more frequent among *Candida*-negative patients, and this was at the border of significance *p* = 0.056. Other diagnoses among *Candida*-negative patients were dyspepsia (17 patients), chronic gastritis (12 patients), duodenal ulcer (9 patients), gastroesophageal reflux (8 patients), hiatus hernia (8 patients), secondary sideropenic anaemia (5 patients) and gastroduodenitis (2 patients). A Chi-square analysis revealed no difference between *Candida*-positive and negative participants regarding the incidence of ulcers (*p*> 0.05).

### 3.4. Statistical Assessment of Association between Detected H. pylori Gene Mutations Associated with Antibiotic Resistance and Demographic, Clinical and Histological Parameters

The presence of gene mutations associated with resistance to fluoroquinolones and clarithromycin was assessed for *gyrA*, *23S*, and *gyr87* and *gyr91* codon. Overall, the resistance gene mutations to clarithromycin and fluoroquinolone antibiotic classes were found in 57 (52.8%) and 52 (47.3%) samples, respectively (Figure 1).

Correlation analysis revealed a significant association between the resistance to antibiotics and the number of treatments, with a significantly higher incidence of 23S and gyrA mutations observed in previously treated participants, compared to the naïve group (*p* < 0.05). In particular, significant difference in resistance rates was observed between patients with more than one eradication attempt, compared to the eradication-naïve (naïve) and patients with one episode of eradication, for fluoroquinolone, clarithromycin and dual resistance (*p* < 0.05). Moreover, higher antibiotics resistance rates were confirmed in patients with failed eradication attempts, for fluoroquinolones (*p* = 0.008) and clarithromycin (*p* = 0.002), compared to the naïve group. An overall dual antimicrobial resistance was detected in 33 from 110 (30%) participants. In addition to the higher incidence of detected mutations among participants with recurrent *H. pylori* infection, significantly higher cellularity, inflammation and atrophy scores were found in previously treated patients compared to the naïve participants (*p* < 0.05), as shown in Table 3.

Interestingly, resistance to clarithromycin was associated with fluoroquinolone resistance (*p* = 0.034), as presented in Table 4. Analysis of demographic, clinical and histopathological characteristics in patients separated in groups by their antibiotic resistance status, revealed a significant difference gender-wise, with a higher incidence of clarithromycin-related gene mutations in the female cohort, compared to the male (*p* = 0.008). Women also had a higher incidence of resistance to both drugs (*p* = 0.004). There was no significant difference between antibiotic resistance and other parameters, including age, *Candida* colonization and the presence of gastrointestinal inflammatory diseases (gastritis, duodenitis and ulcers) (*p* > 0.05).

Significantly higher atrophy scores were observed in patients with resistance to fluoroquinolones (*p* = 0.001) and dual resistance (*p* = 0.007), compared to the patients with fluoroquinolone-sensitive *H. pylori*. No significant difference was observed for the resistance gene mutations and other pathohistological criteria (*p* > 0.05).

## 4. Discussion

Considering an increasing prevalence of *H. pylori* infection, numerous studies have been conducted in order to elucidate factors associated with its eradication. Traditional factors such as patients’ compliance due to therapy duration, as well as potential adverse effects of applied regimen, have been thoroughly evaluated [33]. *H. pylori’s* resistance represents a major factor in the failure of eradication therapy, and the worldwide increase in the prevalence of resistant strains at an alarming rate has challenged the commonly used triple therapy approach [8,12,34,35]. Although the prevalence of resistant *H. pylori* strains varies widely across different geographic regions, an association with socioeconomic status has been observed, with higher rates in developing countries [3,36]. A global decrease in the eradication rate has been observed, with <75% of successful treatments commonly reported in the studies [36]. In the majority of developed countries, resistant *H. pylori* strains to clarithromycin, metronidazole and levofloxacin were assessed to be more than 15%, with clarithromycin resistance the most commonly associated with the treatment failure [37]. The clarithromycin-resistant *H. pylori* was recently recognized by WHO as a high-priority pathogen for investment in new therapeutics [38,39]. Our alarming data suggested a high resistance to clarithromycin and levofloxacin and further support these studies. Molecular mechanisms behind *H. pylori* resistance to antibiotics have been intensively researched and point mutations have been identified as the major cause of antibiotics failure [12]. Studies revealed that point mutations in domain V of the 23S rRNA gene and several positions in subunit A of DNA gyrase (*gyrA*, *gyr87,* and *gyr91)* are responsible for *H. pylori’s* resistance to macrolide and quinolone antibiotics, respectively [8,12,40,41]. The investigation of the common mutations associated with resistance to antibiotics in the present study revealed a significantly higher prevalence of antibiotics resistance genes in subjects who previously had a course of anti-*H. pylori* antibiotics. The analysis of the correlation between the antibiotic resistance to clarithromycin and fluoroquinolone and the eradication status revealed a significantly higher frequency of mutations related to resistance in previously treated subjects, compared with the untreated group, *p* < 0.05, as presented in Table 3.

**Figure 2:**
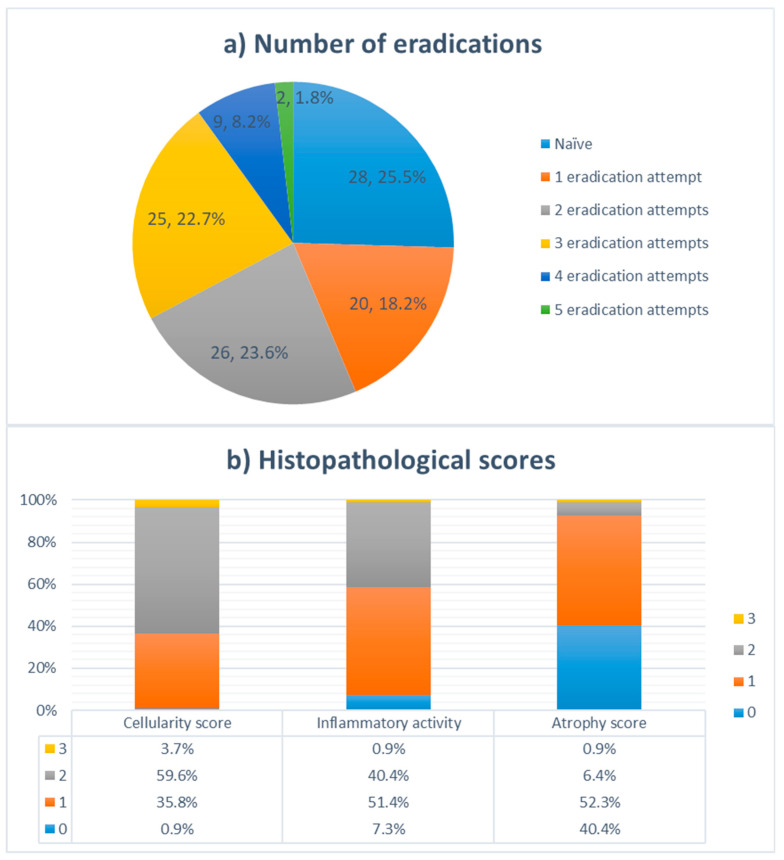
(**a**) Distribution of patients in the cohort according to the number of *H. pylori* eradication attempts (*n*, %); (**b**) Distribution of cellularity, inflammatory activity, and atrophy scores among patients in the cohort.

In 2018, the overall prevalence of antibiotic resistance in the European region was estimated to be 32% and 14% for clarithromycin and levofloxacin, respectively. Resistance to clarithromycin has been characterized by high geographical heterogeneity in resistance rates, ranging from 96% in Australia to 15% in Italy [11,42]. In recently published data from the currently largest registered clinical trial regarding *H. pylori* antimicrobial resistance to take place in the USA and Europe, a comparable and alarmingly high clarithromycin resistance rate was affirmed. Culture based, susceptibility testing revealed that all USA subregions and the majority of European countries have clarithromycin resistance rates above 15% (universally accepted threshold) [14]. Although quinolones had previously been used as an alternative treatment, the rise in *H. pylori* strains resistant to other antibiotics has increased the usage of levofloxacin and other quinolone drugs. Levofloxacin resistance was estimated to be significantly lower compared to clarithromycin, ranging from 5% in Australia and Italy to 15% in France and 16% in Spain [11]. Worryingly, high resistance rates to both antibiotics were observed in the present study, with 57 (51.8%) and 52 (47.3%) participants colonized with *H. pylori* resistant to clarithromycin and fluoroquinolones, respectively, showing a significant increase in resistance to antibiotic drugs (Figure 1). Moreover, 33 (30%) subjects had resistance genes to both antibiotic groups which could be partially explained by the large percentage of patients with more than one eradication attempt (75%, Figure 2). Despite the fact that a small cohort of patients was analyzed here, the alarming prevalence of antibiotic-resistant *H. pylori* strains among Serbian patients requires urgent research and the development of new treatment strategies.

In addition to the primary association of treatment failure with resistance to antibiotics, other factors may further influence the outcome of eradication, including the possible entry of *H. pylori* inside eukaryotic cells [22]. Intracellular *H. pylori* has been confirmed in *Candida* species isolated from oral, gastric and vaginal samples. The internalization of *H. pylori* inside yeast cells has been proposed to be a possible route of transmission, as the yeast cells are more resistant to the stressful environment [16,20,43,44]. *Sanchez-Alonzo* and colleagues discovered several driving factors of this endosymbiotic relationship, that trigger the harboring of *H. pylori* within *Candida* vacuoles, including temperatures outside the optimal growth range for *H. pylori*, antibiotics treatment, unfavorable pH conditions [19,23,45]. Results of another study indicate that the harboring of *H. pylori* within *Candida* is increased in nutrient-deficient medium and that the endosymbiotic relationship is highly dependent on the *H. pylori* strain involved [46]. The development of molecular techniques allows researchers an expanded and more accurate insight into the composition of the gastric microbial communities, although very few studies focus on studying fungi and their interrelationship with *H. pylori* and other members of gastric mucosa remain under-researched [47]. To our knowledge, this is the first study where the presence of *Candida* yeasts was detected, and *Candida* species were identified using the culture-independent, RT-PCR method in patients with confirmed *H. pylori* infection. Previously, Karczewska et al., who studied the co-existence of *Candida* and *H. pylori* by using cultivation techniques, reported that 11% of the patients who underwent endoscopy harbored both microorganisms [21]. Similar to the results of cultivation-based study, in our molecular assessment, the presence of both *Candida* and *H. pylori* was confirmed in nine patients (8.18%). The most prevalent *Candida* species was *C. albicans* found in five *Candida*-positive patients, followed by *C. tropicalis* and *C. parapsilosis* identified in two patients each. Previous studies have identified *C. albicans* as the most frequently isolated fungus from the gastric mucosa while other *Candida* species have also been detected, including *C. glabrata*, *C. parapsilosis*, *C. famata* and *C. tropicalis* [29,48].

The majority of patients in our study had been diagnosed with gastritis or duodenitis (*n* = 74, 67.3%), which provides further evidence of *H. pylori* as a causative factor in gastritis development [49]. While *Karczewska* and colleagues reported a significantly higher incidence of gastric ulcers in the group with *H. pylori* and *Candida* co-infection [21], no statistical difference in the incidence of ulcers was found among *Candida*-positive and negative patients in the current study (*p* > 0.05). The present results indicate a lower frequency of gastritis or duodenitis in a group with detected *Candida* colonization at the border of significance (*p* = 0.056); it should be noted that a relatively small number of *Candida*-positive samples hampered a reliable statistical assessment. Compared to the study conducted by *Zwolińska* and colleagues, where fungal colonization was detected in 54.2% of participants with gastric ulcers and 10.3% of chronic gastritis cases, we observed a much lower incidence of gastric ulcers (*n* = 1; 11%) in *Candida*-positive patients [29]. GERD was the most frequently diagnosis among *Candida*-positive patients in our cohort (*n* = 3; 33%), followed by dyspepsia and gastroduodenitis (*n* = 2; 22%), representing the variety of gastric pathologies connected with *H. pylori* colonization [12,50].

*Candida-*positive patients had a significantly older age (*p* < 0.05), which further supports previous findings that older age is a major predisposing factor for the fungal colonization of the stomach [51]. According to the current *Maastricht* guidelines, a high dose of PPI is recommended for *H. pylori* eradication therapy and all *H. pylori*-positive patients should be treated with antibiotics [7,45]; meanwhile, it has been shown that in addition to the older age, treatment with PPIs also favors *Candida* colonization of the gastric mucosa [51]. Besides representing part of the therapy regimens for *H. pylori* eradication, PPIs are widely prescribed drugs for other indications. However, recent studies have proved that chronic PPI usage promotes perturbations in gastric microbial communities by altering the pH value in the stomach [48,52]. A connection between PPI usage and fungal colonization of the stomach has been also confirmed in a study by *Mottaghi* and colleagues, where the use of ranitidine, pantoprazole and omeprazole was associated with a significantly increased risk of gastric candidiasis [31].

Previous studies indicated a connection between *H. pylori* infection and intestinal microbiota dysbiosis [25]. The alterations in the fungal gastrointestinal community, characterized by an increased abundance of *Candida glabrata* and other unclassified fungi, were also found to be associated with *H. pylori* infection [53]. In addition to the direct impact that *H. pylori* on gastric microbial communities, attempts at *H. pylori* eradication using several courses of antibiotics also affect the gastric microbiota and may result in gastric dysbiosis [54,55]. In this analysis’ cohort, all patients were *H. pylori* positive. We found no significant difference in resistance rates between *Candida*-positive and *Candida*-negative groups, indicating that yeast colonization alone does not have a major impact on the transmission of *H. pylori* resistance genes.

In addition to the endosymbiotic relationship, other forms of interactions between fungal and bacterial gastric community members have been observed, including the adherence of *H. pylori* to *Candida* species and the development of polymicrobial biofilms [56,57]. However, it is still not clear whether the co-infection of *Candida* and *H. pylori* exacerbates the development of gastric diseases. The results of recent studies indicate the importance of other gastric microbial members in the pathogenesis of gastric inflammatory diseases. Notably, the existence of a significant correlation between gastric dysbiosis and aforementioned pathologies was observed, highlighting the importance of perturbations in the gastric microbiome in the etiopathology of gastric diseases [24,27,28,53]. Moreover, an association between dysbiosis of the fungal gastric community and the development of gastritis, ulcers and gastric cancer has been also proven in several studies, indicating the significance of microbiota dysbiosis [47,58,59].

In order to combat a growing global antibiotic resistance and prevalence of *H. pylori* infection, a more responsible antibiotic stewardship should be implemented. Tailored therapy based on individual susceptibility testing has been proved to be more effective than empiric therapy (especially in high clarithromycin resistance regions) [14]. Although regular testing for resistance genes has not been recommended by the last Maastricht guideline, awareness of the strain-specific genetic structure could improve treatment effectiveness, i.e., help to tailor therapy that would reduce the number of eradication attempts, to the benefits of patients and healthcare providers. [7]. Assessing antibiotic susceptibility prior to eradication therapy is a relatively novel therapeutic approach that allows for an individualization of therapy. However, studies have shown that the *H. pylori*-antibiotics interrelationship is more intricate and tailored therapy does not always lead to eradication [60]. This could be, potentially, attributed to *Candida* colonization which would enable resistance to an antibiotic susceptible *H. pylori* strain, by allowing it intracellular protection, leading to treatment failure. Latifi-Navid et al. and our unpublished data reveal ketoconazole as an agent which can additionally lead to successful eradication and can reduce the chance of recurrence of bacterial infection. However, further studies are warranted to elucidate the extent of their relationship [61].

## 5. Conclusions

In this study, antibiotic resistance genes and colonization with *Candida* yeast was assessed in a small cohort of *H. pylori*-positive patients, with an idea to elucidate reasons for *H. pylori* eradication failure. While there is an alarming level of antibiotic resistance (52% and 47% of participants possessed genes related to clarithromycin and fluoroquinolone resistance, respectively), the co-occurrence of *H. pylori* and *Candida* species were found in only 8% of patients. In the analyzed cohort, the fungal colonization was associated with older age, suggesting that the age-related changes in physiological functions and microbiome composition favors *Candida* colonization of the stomach. Previous studies suggested that *Candida* could serve as a host facilitating a rescue of *H. pylori* under unfavorable conditions. Our results suggest that such phenomena could exist only in a small proportion of patients. A global increase in the prevalence of resistant *H. pylori* strains has resulted in an increased interest in *H. pylori* infection management strategies. New approaches, including vaccines, phytochemicals, secondary bacterial metabolites and probiotic strains are needed. Moreover, as studies of the mechanisms of *H. pylori* invasiveness and colonization revealed the facultative intracellular nature of bacteria, further research into the endosymbiotic relationship between *H. pylori* and *Candida* species is suggested.

## Figures and Tables

**Table 1:** Temperatures used for *Candida* species identification and number of identified *Candida* species.

Melting Curves Temperatures Used for *Candida* Identification (°C)	*Candida* Species	Number of *Candida–*Positive Patients *n*, (%)
87.2/87.7	*C. albicans*	5 (55.6%)
84.7	*C. tropicalis*	2 (22.2%)
85.3	*C. parapsilosis*	2 (22.2%)

**Table 2:** Overview of characteristics in *Candida*-negative and *Candida*-positive patients.

Categories	*Candida*–Negative Group *n*, (%)	*Candida*–Positive Group *n*, (%)	*p* Value
Number of participants	101 (91.82%)	9 (8.18%)	
Median age (years)	55	66	
<40	20 (19.80%)	0	0.208
40–50	22 (21.80%)	0	
>50	59 (58.40%)	9 (100%)	0.012 *
Gender			0.152
Male	39 (38.60%)	6 (66.70%)	
Female	62 (61.40%)	3 (33.30%)	
Number of eradications			
Naïve	27 (26.70%)	1 (11.1%)	0.443
>= 1 eradications	74, (73.3%)	8 (88.9%)	
>=2 eradications	55, (54.5%)	6 (66.7%)	0.729
Resistance to antibiotics			
Fluoroquinolone (FLQ)	50 (49.5%)	2 (22.2%)	0.167
Clarithromycin (CLA)	52 (51.5%)	5 (55.6%)	1
Dual Resistance (FLQ-R and CLA-R)	32 (31.7%)	1 (11.1%)	0.274
FLQ-S and CLA-S	31 (30.7%)	3 (33.3%)	1
FLQ-R or CLA-R	70 (69.3%)	6 (66.7%)	0.184
Mutations			
gyrA MUT	41 (40.6%)	1 (11.1%)	0.149
23S MUT	51 (50.5%)	5 (55.6%)	1
gyr87 MUT	16 (15.8%)	0	0.352
gyr91 MUT	20 (19.8%)	1 (11.1%)	1
Histopathological categories			
Cellularity score			0.812
0	1 (1.0%)	/	
1	36 (36.0%)	3 (33.0%)	
2	59 (59.0%)	6 (67.0%)	
3	4 (4.0%)	/	
Inflammatory activity			0.554
0	8 (8.0%)	/	
1	50 (50.0%)	6 (66.7%)	
2	41 (41.0%)	3 (33.3%)	
3	1 (1.0%)	/	
Atrophy score			0.601
0	41 (41.0%)	3 (33.3%)	
1	51 (51.0%)	6 (66.7%)	
2	7 (7.0%)	/	
3	1 (1.0%)	/	

* p < 0.05 significance level.

**Table 3:** Overview of characteristics in naïve and previously eradicated patients.

Categories	Total	Treatment Status		*p* Value
		Naïve	Previously Treated	
	*n*, (%)	*n*	*n*	
Fluoroquinolone (FLQ) resistance	52 (47.3%)	7	45	0.008 *
Clarithromycin (CLA) resistance	57 (52.8%)	7	50	0.002 *
Dual Resistance (FLQ-R and CLA-R)	33 (30%)	4	29	0.054
Mutations				
23S mutation	56 (50.9%)	7	49	0.002 *
gyr 91 mutation	21 (19.1%)	3	18	0.268
gyrA mutation	42 (38.2%)	5	37	0.013 *
gyr 87 mutation	16 (14.5%)	3	13	0.757
Histopathological scores				
Atrophy score				0.018 *
0	44	18	26	
1	57	8	49	
2	7	2	5	
3	1	0	1	
Cellularity score				0.018 *
0	1	1	0	
1	39	15	24	
2	65	12	53	
3	4	0	4	
Inflammatory activity				0.002 *
0	8	5	3	
1	56	17	39	
2	44	6	38	
3	1	0	1	

*p < 0.05 significance level.

**Table 4:** Results of Chi-square analysis of characteristics between antibiotic resistant and sensitive groups.

Categories	FLQ Resistance (*p* Value)	CLA Resistance (*p* Value)	Dual Resistance (*p* Value)
>40 years old	0.636	1	0.787
>50 years old	0.889	0.918	0.638
Gender	0.094	0.008 *	0.004 *
*Candida* status	0.222	0.815	0.362
Number of eradication	0.001 *	0.003 *	0.001 *
Naïve vs Repeated Eradication	0.012 *	0.001 *	0.062
Naïve and 1 vs. >1 eradication	0.001 *	0.001 *	0.001 *
Celularity score	0.907	0.333	0.453
Celularity score 0 and 1 vs. >1	0.628	0.246	0.259
Inflammatory activity	0.336	0.73	0.242
Inflammatory activity 0 and 1 vs. >1	0.34	0.053	0.223
Atrophy score	0.001 *	0.097	0.007 *
Atrophy score 0 vs. >0	0.017 *	0.078	0.013 *
Gastritis/Duodenitis	0.833	0.639	0.562
Ulcers	0.295	0.462	0.237
Fluoroquinolone (FLQ) Resistance		0.034 *	

* p < 0.05 significance level.

## Data Availability

Data regarding investigated patents and statistical analysis will not be made publicly available.

## References

[1] Kotilea K., Bontems P., Touati E., Kamiya S., Backert S. (2019). Epidemiology, Diagnosis and Risk Factors of *Helicobacter pylori* Infection. Helicobacter pylori in Human Diseases: Advances in Microbiology, Infectious Diseases and Public Health.

[2] Marcus E.A., Scott D.R., Kim N. (2016). Gastric Colonization by *H. pylori*. Helicobacter pylori.

[3] Sjomina O., Pavlova J., Niv Y., Leja M. (2018). Epidemiology of *Helicobacter pylori* infection. Helicobacter.

[4] Zamani M., Ebrahimtabar F., Zamani V., Miller W.H., Alizadeh-Navaei R., Shokri-Shirvani J., Derakhshan M.H. (2018). Systematic review with meta-analysis: The worldwide prevalence of *Helicobacter pylori* infection. Aliment. Pharmacol. Ther..

[5] Marshall B.J., Warren J.R. (1984). Unidentified curved bacilli in the stomach of patients with gastritis and peptic ulceration. Lancet.

[6] Moss S.F. (2017). The Clinical Evidence Linking *Helicobacter pylori* to Gastric Cancer. Cell Mol. Gastroenterol. Hepatol..

[7] Malfertheiner P., Megraud F., Rokkas T., Gisbert J.P., Liou J.M., Schulz C., Gasbarrini A., Hunt R.H., Leja M., O’Morain C. (2022). Management of *Helicobacter pylori* infection: The Maastricht VI/Florence consensus report. Gut.

[8] Francesco V.D., Zullo A., Hassan C., Giorgio F., Rosania R., Ierardi E. (2011). Mechanisms of *Helicobacter pylori* Antibiotic Resistance: An updated appraisal. World J. Gastrointest. Pathophysiol..

[9] Nyssen O.P., Bordin D., Tepes B., Pérez-Aisa Á., Vaira D., Caldas M., Bujanda L., Castro-Fernandez M., Lerang F., Leja M. (2021). European Registry on *Helicobacter pylori* management (Hp-EuReg): Patterns and trends in first-line empirical eradication prescription and outcomes of 5 years and 21 533 patients. Gut.

[10] Nyssen O.P., Vaira D., Pérez-Aísa Á., Rodrigo L., Castro-Fernandez M., Jonaitis L., Tepes B., Vologzhanina L., Caldas M., Lanas A. (2022). Empirical second-line therapy in 5000 patients of the European registry on *Helicobacter pylori* management (Hp-EuReg). Clin. Gastroenterol. Hepatol..

[11] Savoldi A., Carrara E., Graham D.Y., Conti M., Tacconelli E. (2018). Prevalence of Antibiotic Resistance in *Helicobacter pylori*: A Systematic Review and Meta-analysis in World Health Organization Regions. Gastroenterology.

[12] Tshibangu-Kabamba E., Yamaoka Y. (2021). *Helicobacter pylori* infection and antibiotic resistance—From biology to clinical implications. Nat. Rev. Gastroenterol. Hepatol..

[13] Boyanova L., Hadzhiyski P., Kandilarov N., Markovska R., Mitov I. (2019). Multidrug resistance in *Helicobacter pylori*: Current state and future directions. Expert Rev. Clin. Pharmacol..

[14] Mégraud F., Graham D.Y., Howden C.W., Trevino E., Weissfeld A., Hunt B., Smith N., Leifke E., Chey W.D. (2022). Rates of Antimicrobial Resistance in *Helicobacter pylori* Isolates from Clinical Trial Patients Across the US and Europe. Am. J. Gastroenterol..

[15] Morino Y., Sugimoto M., Nagata N., Niikiura R., Iwata E., Hamada M., Kawai Y., Fujimiya T., Takeuchi H., Unezaki S. (2021). Influence of Cytochrome P450 2C19 Genotype on *Helicobacter pylori* Proton Pump Inhibitor-Amoxicillin-Clarithromycin Eradication Therapy: A Meta-Analysis. Front. Pharmacol..

[16] Hou C., Yin F., Wang S., Zhao A., Li Y., Liu Y. (2022). *Helicobacter pylori* Biofilm-Related Drug Resistance and New Developments in Its Anti-Biofilm Agents. Infect. Drug Resist..

[17] Campos P.L., Merino-Barrera J.S., Smith C.T., García-Cancino A. (2018). *Candida* sp. as a potential reservoir and transmission facilitator of *Helicobacter pylori*. Biomed. J. Sci. Tech. Res..

[18] Siavoshi F., Saniee P. (2014). Vacuoles of Candida yeast as a specialized niche for *Helicobacter pylori*. World J. Gastroenterol..

[19] Sánchez-Alonzo K., Arellano-Arriagada L., Castro-Seriche S., Parra-Sepúlveda C., Bernasconi H., Benavidez-Hernández H., Campos V.L., Sáez K., Smith C.T., García-Cancino A. (2021). Temperatures Outside the Optimal Range for *Helicobacter pylori* Increase Its Harboring within *Candida* Yeast Cells. Biology.

[20] Saniee P., Siavoshi F., Nikbakht-Broujeni G., Khormali M., Sarrafnejad A., Malekzadeh R. (2013). Localization of *H. pylori* within the vacuole of Candida yeast by direct immunofluorescence technique. Arch. Iran Med..

[21] Karczewska E., Wojtas I., Sito E., Trojanowska D., Budak A., Zwolinska-Wcislo M., Wilk A. (2009). Assessment of co-existence of *Helicobacter pylori* and Candida fungi in diseases of the upper gastrointestinal tract. J. Physiol. Pharmacol..

[22] Mason K.L., Erb-Downward J.R., Falkowski N.R., Young V.B., Kao J.Y., Huffnagle G.B. (2012). Interplay between the gastric bacterial microbiota and Candida albicans during postantibiotic recolonization and gastritis. Infect. Immun..

[23] Sánchez-Alonzo K., Belmar L., Parra-Sepúlveda C., Bernasconi H., Campos V.L., Smith C.T., Sáez K., García-Cancino A. (2021). Antibiotics as a Stressing Factor Triggering the Harboring of *Helicobacter pylori* J99 within Candida albicans ATCC10231. Pathogens.

[24] Duan X., Chen P., Xu X., Han M., Li J. (2022). Role of Gastric Microorganisms Other than *Helicobacter pylori* in the Development and Treatment of Gastric Diseases. BioMed Res. Int..

[25] Rajilic-Stojanovic M., Figueiredo C., Smet A., Hansen R., Kupcinskas J., Rokkas T., Andersen L., Machado J.C., Ianiro G., Gasbarrini A. (2020). Systematic review: Gastric microbiota in health and disease. Aliment. Pharmacol. Ther..

[26] Nardone G., Compare D. (2015). The human gastric microbiota: Is it time to rethink the pathogenesis of stomach diseases?. United Eur. Gastroenterol. J..

[27] Hansen A., Johannesen T.B., Spiegelhauer M.R., Kupcinskas J., Urba M., Skieceviciene J., Jonaitis L., Frandsen T.H., Kupcinskas L., Fuursted K. (2020). Distinct composition and distribution of the gastric mycobiota observed between dyspeptic and gastric cancer patients evaluated from gastric biopsies. Microb. Heal. Dis..

[28] Ianiro G., Molina-Infante J., Gasbarrini A. (2015). Gastric Microbiota. Helicobacter.

[29] Zwolinska-Wcisło M., Budak A., Bogdał J., Trojanowska D., Stachura J. (2001). Fungal colonization of gastric mucosa and its clinical relevance. Med. Sci. Monit..

[30] Dixon M.F., Genta R.M., Yardley J.H., Correa P. (1996). Classification and grading of gastritis. The updated Sydney System. International Workshop on the Histopathology of Gastritis, Houston 1994. Am. J. Surg. Pathol..

[31] Guimarães N., Azevedo N.F., Figueiredo C., Keevil C.W., Vieira M.J. (2007). Development and application of a novel peptide nucleic acid probe for the specific detection of *Helicobacter pylori* in gastric biopsy specimens. J. Clin. Microbiol..

[32] Zhang J., Hung G.C., Nagamine K., Li B., Tsai S., Lo S.C. (2016). Development of Candida-Specific Real-Time PCR Assays for the Detection and Identification of Eight Medically Important Candida Species. Microbiol. Insights.

[33] Lü M., Yu S., Deng J., Yan Q., Yang C., Xia G., Zhou X. (2016). Efficacy of probiotic supplementation therapy for *Helicobacter pylori* eradication: A meta-analysis of randomized controlled trials. PLoS ONE.

[34] Thung I., Aramin H., Vavinskaya V., Gupta S., Park J.Y., Crowe S.E., Valasek M.A. (2016). Review article: The global emergence of *Helicobacter pylori* antibiotic resistance. Aliment. Pharmacol. Ther..

[35] Zou Y., Qian X., Liu X., Song Y., Song C., Wu S., An Y., Yuan R., Wang Y., Xie Y. (2020). The effect of antibiotic resistance on *Helicobacter pylori* eradication efficacy: A systematic review and meta-analysis. Helicobacter.

[36] Jaka H., Rhee J.A., Östlundh L., Smart L., Peck R., Mueller A., Kasang C., Mshana S.E. (2018). The magnitude of antibiotic resistance to *Helicobacter pylori* in Africa and identified mutations which confer resistance to antibiotics: Systematic review and meta-analysis. BMC Infect. Dis..

[37] Perna F., Zullo A., Ricci C., Hassan C., Morini S., Vaira D. (2007). Levofloxacin-based triple therapy for *Helicobacter pylori* re-treatment: Role of bacterial resistance. Dig. Liver Dis..

[38] Tacconelli E., Carrara E., Savoldi A., Harbarth S., Mendelson M., Monnet D.L., Pulcini C., Kahlmeter G., Kluytmans J., Carmeli Y. (2018). Discovery, research, and development of new antibiotics: The WHO priority list of antibiotic-resistant bacteria and tuberculosis. Lancet Infect. Dis..

[39] Sasaki K. (2012). Candida-associated gastric ulcer relapsing in a different position with a different appearance. World J. Gastroenterol..

[40] Marques A.T., Vítor J.M.B., Santos A., Oleastro M., Vale F.F. (2020). Trends in *Helicobacter pylori* resistance to clarithromycin: From phenotypic to genomic approaches. Microb. Genom..

[41] Talarico S., Korson A.S., Leverich C.K., Park S., Jalikis F.G., Upton M.P., Broussard E., Salama N.R. (2018). High prevalence of *Helicobacter pylori* clarithromycin resistance mutations among Seattle patients measured by droplet digital PCR. Helicobacter.

[42] Ghotaslou R., Leylabadlo H.E., Asl Y.M. (2015). Prevalence of antibiotic resistance in *Helicobacter pylori*: A recent literature review. World J. Methodol..

[43] Sánchez-Alonzo K., Matamala-Valdés L., Parra-Sepúlveda C., Bernasconi H., Campos V.L., Smith C.T., Sáez K., García-Cancino A. (2021). Intracellular Presence of *Helicobacter pylori* and Its Virulence-Associated Genotypes within the Vaginal Yeast of Term Pregnant Women. Microorganisms.

[44] Sánchez-Alonzo K., Parra-Sepúlveda C., Vergara L., Bernasconi H., García-Cancino A. (2020). Detection of *Helicobacter pylori* in oral yeasts from students of a Chilean university. Rev. Assoc. Med. Bras..

[45] Sánchez-Alonzo K., Parra-Sepúlveda C., Vega S., Bernasconi H., Campos V.L., Smith C.T., Sáez K., García-Cancino A. (2020). In vitro incorporation of *Helicobacter pylori* into Candida albicans caused by acidic pH stress. Pathogens.

[46] Sánchez-Alonzo K., Silva-Mieres F., Arellano-Arriagada L., Parra-Sepúlveda C., Bernasconi H., Smith C.T., Campos V.L., García-Cancino A. (2021). Nutrient Deficiency Promotes the Entry of *Helicobacter pylori* Cells into Candida Yeast Cells. Biology.

[47] Wang Z., Ren R., Yang Y. (2020). Mucosa microbiome of gastric lesions: Fungi and bacteria interactions. Prog. Mol. Biol. Transl. Sci..

[48] Mottaghi B., Emami M.H., Riahi P., Fahim A., Rahimi H., Mohammadi R. (2021). Candida colonization of the esophagus and gastric mucosa; a comparison of patients taking proton pump inhibitors and those taking histamine receptor antagonist drugs. Gastroenterol. Hepatol. Bed Bench.

[49] Sugano K., Tack J., Kuipers E.J., Graham D.Y., El-Omar E.M., Miura S., Haruma K., Asaka M., Uemura N., Malfertheiner P. (2015). Kyoto global consensus report on *Helicobacter pylori* gastritis. Gut.

[50] Tham K.T., Peek R.M., Atherton J.C., Cover T.L., Perez-Perez G.I., Shyr Y., Blaser M.J. (2001). *Helicobacter pylori* genotypes, host factors, and gastric mucosal histopathology in peptic ulcer disease. Hum. Pathol..

[51] Massarrat S., Saniee P., Siavoshi F., Mokhtari R., Mansour-Ghanaei F., Khalili-Samani S. (2016). The Effect of *Helicobacter pylori* Infection, Aging, and Consumption of Proton Pump Inhibitor on Fungal Colonization in the Stomach of Dyspeptic Patients. Front. Microbiol..

[52] Ierardi E., Losurdo G., Fortezza R.F., Principi M., Barone M., Leo A.D. (2019). Optimizing proton pump inhibitors in *Helicobacter pylori* treatment: Old and new tricks to improve effectiveness. World J. Gastroenterol..

[53] Dash N.R., Khoder G., Nada A.M., Al Bataineh M.T. (2019). Exploring the impact of *Helicobacter pylori* on gut microbiome composition. PLoS ONE.

[54] Ianiro G., Tilg H., Gasbarrini A. (2016). Antibiotics as deep modulators of gut microbiota: Between good and evil. Gut.

[55] Chen C.-C., Liou J.M., Lee Y.C., Hong T.C., El-Omar E.M., Wu M.S. (2021). The interplay between *Helicobacter pylori* and gastrointestinal microbiota. Gut Microbes.

[56] Ansorg R., Schmid E.N. (1998). Adhesion of *Helicobacter pylori* to yeast cells. Zent. Für Bakteriol..

[57] Palencia S.L., García A., Palencia M. (2022). Multiple surface interaction mechanisms direct the anchoring, co-aggregation and formation of dual-species biofilm between Candida albicans and *Helicobacter pylori*. J. Adv. Res..

[58] Yang P., Zhang X., Xu R., Adeel K., Lu X., Chen M., Shen H., Li Z., Xu Z. (2022). Fungal Microbiota Dysbiosis and Ecological Alterations in Gastric Cancer. Front. Microbiol..

[59] Papon N., Hohl T.M., Zhai B. (2021). Mycobiota dysbiosis and gastric tumorigenesis. Theranostics.

[60] Gisbert J.P. (2020). Empirical or susceptibility-guided treatment for *Helicobacter pylori* infection? A comprehensive review. Ther. Adv. Gastroenterol..

[61] Latifi-Navid S., Siavoshi F., Safari F., Malekzadeh R., Massarrat S. (2006). Antimicrobial effectiveness of ketoconazole against metronidazole-resistant *Helicobacter pylori* isolates from Iranian dyspeptic patients. J. Antimicrob. Chemother..

